# Acupuncture for ovulation induction in polycystic ovary syndrome: study protocol for a multicenter, randomized, sham-controlled trial

**DOI:** 10.3389/fendo.2026.1893429

**Published:** 2026-08-05

**Authors:** Jiashan Li, Zhaohui Li, Lixia Pei, Jingqing Sun, Jian Wang, Qun Lu, Li Yang, Tian Hang, Lingyu Qi, Quanying Zhang, Dongxue An, Chenchen Su, Ge Lu, Xinyu Yan, Yigong Fang, Huanfang Xu

**Affiliations:** 1 Institute of Acupuncture and Moxibustion, China Academy of Chinese Medical Sciences, Beijing, China; 2 Hospital of Acupuncture and Moxibustion, China Academy of Chinese Medical Sciences, Beijing, China; 3 Hohhot First Hospital, Hohhot, China; 4 Jiangsu Province Hospital of Chinese Medicine, Nanjing, China; 5 Beijing Hospital of Traditional Chinese Medicine, Capital Medical University, Beijing, China; 6 The Affiliated Hospital of Shandong University of Traditional Chinese Medicine, Jinan, China; 7 Beijing Chao-Yang Hospital, Capital Medical University, Beijing, China

**Keywords:** acupuncture, ovulation induction, polycystic ovary syndrome, randomized controlled trial, sham acupuncture, study protocol

## Abstract

**Introduction:**

Polycystic ovary syndrome (PCOS) is a common endocrine disorder affecting up to 15% of reproductive-aged women, and is characterized by chronic anovulation that leads to irregular menstrual cycles, infertility, and long-term health risks. While acupuncture has been used for ovulation induction in PCOS, existing evidence remains inconclusive due to methodological limitations and clinical heterogeneity of enrolled populations, which have been largely overlooked. This trial aims to evaluate the efficacy of manual acupuncture for improving ovulation in a precisely defined, more homogeneous PCOS subgroup.

**Methods and analysis:**

This is a prospective, multicenter, randomized, sham-controlled trial. A total of 156 participants with PCOS will be randomly assigned at a 1:1 ratio to receive either manual acupuncture (MA) or sham acupuncture (SA). Eligible participants present with spontaneous menstrual cycles lasting 35–60 days and a body mass index (BMI) below 28.0 kg/m², and this trial targets a subgroup of patients with mild-to-moderate ovulatory dysfunction. The intervention lasts 12 weeks and comprises 36 treatment sessions (three sessions weekly), after which participants will enter a 12-week follow-up phase. The primary outcome is the cycle ovulation rate (COR) at Week 12, verified via transvaginal ultrasound. Secondary outcomes include menstrual cycle characteristics, reproductive hormone profile, metabolic parameters, anthropometric measures, quality of life, anxiety and depression, patient global impression of change, and safety outcomes. Intention-to-treat analysis will be performed, and a p-value <0.05 (two-sided) will be defined as the threshold for statistical significance.

**Systematic Review registration:**

https://itmctr.ccebtcm.org.cn/mgt/project/, identifier ITMCTR2025000078

## 1 Introduction

Polycystic ovary syndrome (PCOS) is one of the most prevalent endocrine disorders in reproductive-aged women, affecting approximately 5–15% of this population (1). It is characterized by marked clinical heterogeneity, as confirmed by recent large-scale data-driven studies (2, 3). Its diagnosis relies on the Rotterdam criteria, which require the presence of at least two of the following three phenotypes: oligo-anovulation, hyperandrogenism, and polycystic ovarian morphology (PCOM) (4). Notably, the 2023 international evidence-based guideline recognizes serum anti-Müllerian hormone (AMH) concentrations as a validated alternative to ultrasound for defining PCOM (1). Chronic anovulation, the core pathophysiological feature of PCOS, leads to irregular menstrual cycles, inadequate endometrial shedding, impaired fertility, and an elevated long-term risk of endometrial hyperplasia. Women with PCOS frequently experience anovulation or irregular ovulation, significantly impairing their fertility, with an estimated 30–40% of affected women seeking help for infertility primarily due to ovulatory dysfunction (5). Recovering ovulatory function thus represents a core therapeutic target and an essential prerequisite for spontaneous conception. Recently, an international consensus has renamed PCOS as polyendocrine metabolic ovarian syndrome (PMOS) to better reflect its multisystem nature (6). Beyond the reproductive complications, PCOS also confers long-term metabolic and cardiovascular risks, raises healthcare resource consumption, and imposes considerable psychological distress stemming from its clinical manifestations (7).

Current first-line pharmacological treatments for ovulation induction, such as clomiphene citrate and letrozole, are widely prescribed, although their use may be limited by side effects, contraindications, or patient preference for non-pharmacological approaches (8, 9). While lifestyle intervention constitutes the cornerstone of PCOS management, sustained long-term compliance poses a major clinical barrier (10). Consequently, there is growing interest in complementary therapies like acupuncture, which is hypothesized to improve ovulatory function in women with PCOS by modulating the hypothalamic-pituitary-ovarian (HPO) axis (11, 12) and regulating autonomic nervous system activity (13, 14).

Published randomized controlled trials (RCTs) evaluating acupuncture for PCOS-related ovulation induction have produced conflicting and inconclusive results. Prior systematic reviews have concluded that while acupuncture may improve ovulation and menstrual frequency in PCOS, the overall evidence is limited by low methodological quality (15, 16). Discrepant outcomes across independent trials further highlight this clinical ambiguity. Several positive trials enrolled more homogeneous PCOS populations through strict eligibility criteria (e.g., body mass index, hormonal profiles) (17–19). In contrast, the large, well-powered factorial RCT by Wu et al. (2017) (20) detected no significant ovulatory benefit of active over control acupuncture; this neutral outcome was replicated in a separate sham-controlled RCT (21). Notably, Subgroup secondary analyses of the Wu et al. trial further revealed heterogeneous treatment responses across subgroups, suggesting that clinical phenotypic heterogeneity acts as a critical effect modifier (22). Taken together, these data indicate that acupuncture may generate superior ovulatory improvements among clearly stratified PCOS subgroups. To provide a definitive evaluation of efficacy, we conducted a prospective, multicenter, randomized, sham-controlled trial. This study integrates robust methodology with a precisely defined study population to control for both non-specific therapeutic context and clinical heterogeneity, thereby offering a conclusive assessment of whether manual acupuncture can restore ovulation in a targeted patient cohort. This investigation aims to contribute to a more refined understanding of acupuncture’s potential role in PCOS management.

## 2 Materials and methods

### 2.1 Study design

This is a multicenter, randomized, sham-controlled trial. The design of this protocol adheres to the Standard Protocol Items: Recommendations for Interventional Trials (SPIRIT) guidelines (23). Descriptions of the manual acupuncture and sham acupuncture interventions comply with the Standards for Reporting Interventions in Controlled Trials of Acupuncture (STRICTA) (24) and the Sham Acupuncture Reporting (SHARE) recommendations (25), respectively. Future reporting of the trial results will follow the Consolidated Standards of Reporting Trials (CONSORT) statement (26).

**Figure 1:**
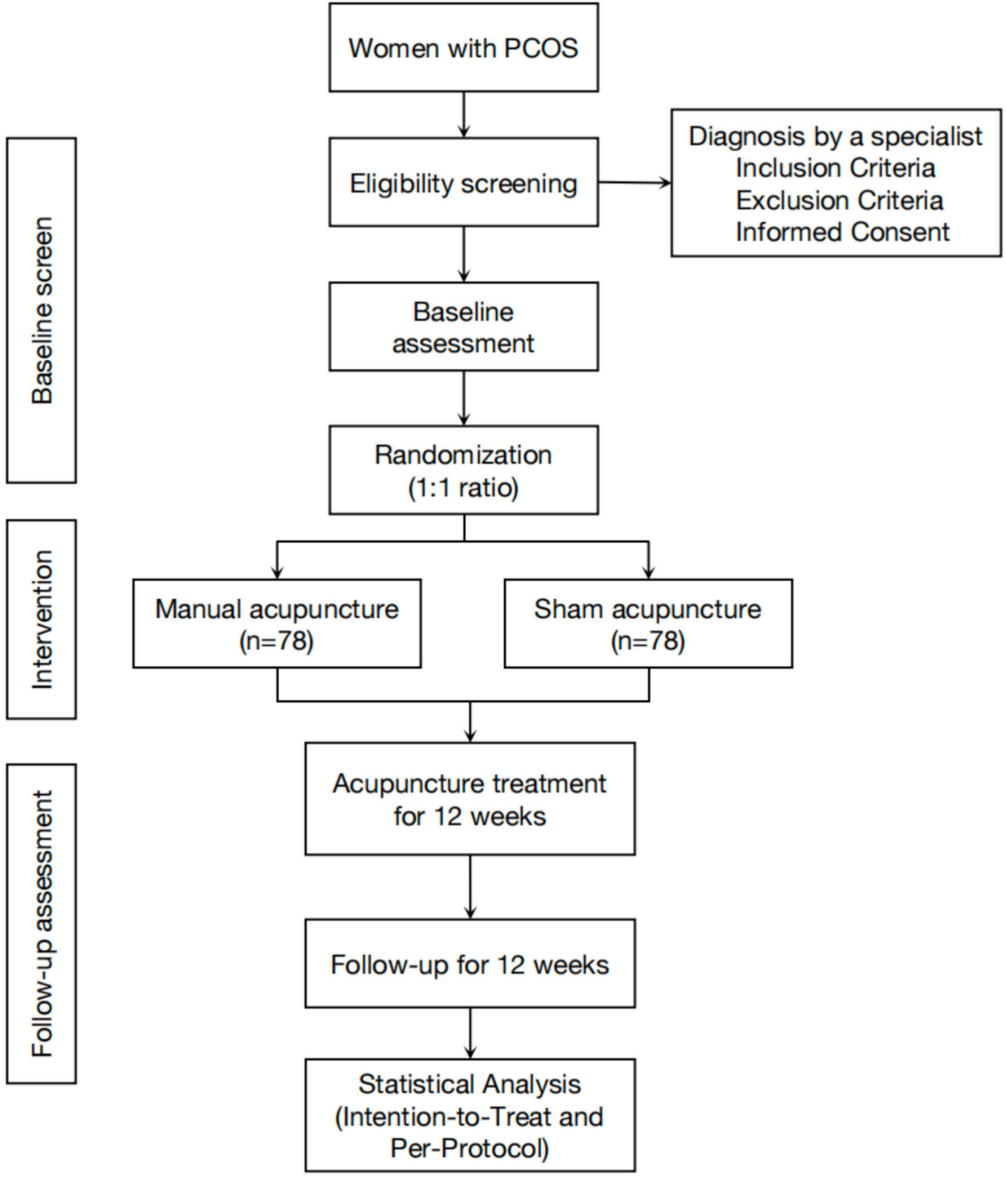
Flow diagram of the trial. PCOS, polycystic ovary syndrome; MA, manual acupuncture; SA, sham acupuncture; ITT, intention-to-treat; PP, per-protocol.

The trial has been registered with the International Traditional Medicine Clinical Trial Registry (registration ID: ITMCTR2025000078). The study protocol and informed consent documents have been reviewed and approved by the Ethics Committee of the China Academy of Chinese Medical Sciences (Approval No.:2024XLW006-2). All participants will provide written informed consent prior to any study-related procedures. All procedures and time frames are displayed in **Figure 1**. In addition, the schedule of enrollment, interventions, and assessments during the study period is shown in **Table 1**.

**Table 1:** Schedule of enrollment, interventions, and assessments.

Study period	Screening	Baseline (Week 0)	Intervention (Weeks 1-12)	Post-treatment (Week 12)	Follow-up (Week 24)
Enrollment					
Eligibility Screening	X				
Informed Consent	X				
Randomization		X			
Interventions					
Manual Acupuncture			3 sessions/week		
Sham Acupuncture			3 sessions/week		
Assessments					
Cycle Ovulation Rate				X	X
Menstrual Calendar*	X	X		X	X
Reproductive Hormones (FAI, LH/FSH)		X		X	X
Metabolic Parameters		X		X	X
Anthropometrics (BMI, WC, HC, WHR)		X		X	X
Questionnaires (PCOSQ, SAS, SDS)		X		X	X
PGIC				X	X
Adverse event			Each visit	X	X
Blinding Assessment				X	
Adherence to treatment				X	X

BMI, body mass index; FAI, free androgen index; FSH, follicle-stimulating hormone; HC, hip circumference; LH, luteinizing hormone; PCOSQ, Polycystic Ovary Syndrome Quality of Life Questionnaire; PGIC, Patient Global Impression of Change; SAS, Self-Rating Anxiety Scale; SDS, Self-Rating Depression Scale; WC, waist circumference; WHR, waist-to-hip ratio.

*At Screening, participants recall their menstrual patterns over the prior 12 months to confirm eligibility (cycle length consistently between 35 and 60 days). At Baseline (Week 0), a paper calendar is provided for prospective daily recording thereafter.

### 2.2 Participants

Participants will be recruited from six clinical centers across China: (1) Hospital of Acupuncture and Moxibustion of the China Academy of Chinese Medical Sciences; (2) Beijing Chao-yang Hospital, Capital Medical University; (3) Beijing Hospital of Traditional Chinese Medicine, Capital Medical University; (4) The Affiliated Hospital of Shandong University of Traditional Chinese Medicine; (5) Jiangsu Province Hospital of Chinese Medicine; and (6) Hohhot First Hospital. The primary recruitment strategy relies on hospital-based referrals: gynecologists and reproductive endocrinologists at each study center screen outpatient attendees and refer potentially eligible candidates during routine clinical visits. To broaden outreach, study advertisements and informational posters will be posted on the official websites, permitted social media platforms (e.g., WeChat), and in the outpatient departments (gynecology, endocrinology, reproductive medicine) of all participating hospitals. Interested candidates can contact the local study coordinator via a dedicated phone line or online questionnaire to complete an initial eligibility prescreen.

### 2.3 Inclusion criteria

Participants will be women diagnosed with polycystic ovary syndrome (PCOS) according to the 2003 Rotterdam criteria (4), after the exclusion of other potential causes of hyperandrogenism or anovulation (e.g., thyroid dysfunction, hyperprolactinemia, congenital adrenal hyperplasia, androgen-secreting tumors). Eligible participants must meet all of the following criteria: (1) aged 21–40 years old; (2) maintained spontaneous menstrual cycles (without hormonal ovulation induction) over the preceding 12 months, with consistent cycle lengths ranging from 35 to 60 days; and (3) provision of written informed consent.

### 2.4 Exclusion criteria

Participants will be excluded if they meet any of the following criteria:

1. Coexisting endocrine or reproductive disorders that could mimic or confound the diagnosis and manifestations of PCOS (e.g., Cushing’s syndrome, congenital adrenal hyperplasia, premature ovarian insufficiency);
2. Major structural or pathological abnormalities of the reproductive tract that could independently affect menstrual cyclicity or fertility (e.g., untreated endometrial polyps, submucosal fibroids, severe intrauterine adhesions, congenital uterine malformations);
3. Use of acupuncture, or any medication/herbal preparation known or suspected to interfere with ovarian function, menstrual cyclicity, or metabolic parameters relevant to PCOS, within the past month;
4. Body mass index (BMI) ≥ 28.0 kg/m²;
5. Currently pregnant or breastfeeding;
6. Coexisting severe systemic diseases (e.g., cardiovascular, hepatic, renal, or psychiatric disorders, malignancy).

### 2.5 Traditional Chinese medicine pattern differentiation criteria

TCM pattern differentiation will be performed by the treating acupuncturist after randomization but prior to the first treatment session. The acupuncturists performing the differentiation are not involved in the randomization process. The diagnostic criteria were developed in accordance with the Criteria of diagnosis and therapeutic effect of gynaenecologic diseases and syndromes in traditional Chinese medicine (ZY/T 001.3-1994) (27) and the General revision and development principle for specification of diagnosis and therapeutic effect evaluation of diseases and syndromes in traditional Chinese medicine (ZY/T 10-2024) (28). Four patterns are predefined: Kidney Deficiency, Phlegm-Dampness, Blood Stasis, and Liver Qi Stagnation. Based on the most prominent clinical presentation, each participant will be assigned to one predominant pattern, with supplementary acupoints added correspondingly as detailed in **Table 2**. Detailed diagnostic criteria are provided in **Supplementary Table 1**.

**Table 2:** Intervention protocol for MA group.

Group & component	Acupoint name (code)	Location (based on WHO standard)	Needling details
A. Core prescription (fixed, alternated by session)			
Set A (Supine)	Zhongwan (CV12)	On the anterior midline, 4 cun superior to the umbilicus.	Perpendicular insertion, 0.5–1.0 cun.
	Huangshu (KI16)	0.5 cun lateral to the umbilicus.	Perpendicular insertion, 0.5–1.0 cun.
	Guanyuan (CV4)	On the anterior midline, 3 cun inferior to the umbilicus.	Perpendicular insertion, 0.5–1.0 cun.
	Dahe (KI12)	0.5 cun lateral to CV4 (Guanyuan).	Perpendicular insertion, 0.5–1.0 cun.
	Zigong (EX-CA1)	3 cun lateral to the midline, 1 cun inferior to CV4.	Perpendicular insertion, 0.5–1.0 cun.
	Zusanli (ST36)	3 cun below ST35, one finger-breadth lateral to tibial crest.	Perpendicular insertion, 0.5–1.2 cun.
	Sanyinjiao (SP6)	3 cun directly above the medial malleolus.	Perpendicular insertion, 0.5–1.0 cun.
Set B (Prone)	Shenshu (BL23)	1.5 cun lateral to the lower border of the L2 spinous process.	Perpendicular insertion, 0.5–1.0 cun.
	Zhongliao (BL33)	In the third posterior sacral foramen.	Oblique insertion toward the foramen, 1.5–2.0 cun.
	Taixi (KI3)	Between the medial malleolus tip and Achilles tendon.	Perpendicular insertion, 0.3–0.5 cun.
B. Standardized adjunct points (added per TCM pattern)			
Kidney Deficiency	Supine: Zhaohai (KI6), Taichong (LR3)	KI6: In depression inferior to medial malleolus. LR3: On dorsum of foot, between 1st and 2nd metatarsals.	Perpendicular insertion, 0.3–0.5 cun.
	Prone: Mingmen (GV4), Ganshu (BL18)	GV4: Below spinous process of L2. BL18: 1.5 cun lateral to lower border of T9 spinous process.	Perpendicular insertion, 0.5–0.8 cun.
Phlegm-Dampness	Supine: Fenglong (ST40), Yinlingquan (SP9)	ST40: 8 cun superior to lateral malleolus, 2 finger-breadth lateral to tibia. SP9: In depression inferior to medial condyle of tibia.	Perpendicular insertion, 0.5–1.2 cun.
	Prone: Pishu (BL20), Weishu (BL21)	BL20: 1.5 cun lateral to lower border of T11 spine. BL21: 1.5 cun lateral to lower border of T12 spine.	Perpendicular insertion, 0.5–0.8 cun.
Blood Stasis	Supine: Hegu (LI4), Xuehai (SP10)	LI4: On dorsum of hand, between 1st and 2nd metacarpals. SP10: 2 cun above medial border of patella.	Perpendicular insertion, 0.5–0.8 cun.
	Prone: Xinshu (BL15), Geshu (BL17)	BL15: 1.5 cun lateral to lower border of T5 spine. BL17: 1.5 cun lateral to lower border of T7 spine.	Perpendicular insertion, 0.5–0.8 cun.
Liver Qi Stagnation	Supine: Ligou (LR5), Taichong (LR3)	LR5: 5 cun above medial malleolus, on posterior border of tibia. LR3: On dorsum of foot, between 1st and 2nd metatarsals.	Perpendicular insertion, 0.5–0.8 cun.
	Prone: Ganshu (BL18), Danshu (BL19)	BL18: 1.5 cun lateral to lower border of T9 spine. BL19: 1.5 cun lateral to lower border of T10 spine.	Perpendicular insertion, 0.5–0.8 cun.

To ensure the reproducibility of TCM pattern differentiation, all acupuncturists will undergo a 2-day centralized training workshop covering the diagnostic criteria for the four predefined patterns, followed by a certification examination (≥80% accuracy required).

### 2.6 Randomization, allocation and blinding

Eligible participants will be randomly assigned at a 1:1 ratio to either the manual acupuncture (MA) group or the sham acupuncture (SA) group. Randomization will be stratified by the six participating clinical centers to ensure baseline balance across groups. An independent statistician will generate the randomization sequence using SAS software (version 9.4), with randomly permuted block sizes of 2 and 4. This sequence will be implemented and managed through a centralized, web-based randomization system maintained by an independent clinical research organization (CRO), ensuring allocation concealment. After obtaining written informed consent and confirming final eligibility, the site investigator will access the central system. The system will then automatically assign a unique trial identifier and the corresponding treatment allocation (MA or SA). The allocation remains fully concealed from both the investigator and the participant until the moment of assignment, thereby preventing selection bias. Owing to the nature of the interventions, the acupuncturists administering the treatments cannot be blinded. Therefore, this is an assessor- and participant-blinded trial. All efforts will be made to maintain blinding of the participants, outcome assessors, and data analysts throughout the study.

### 2.7 Intervention

All acupuncture treatments will be administered by licensed acupuncturists with a minimum of 2 years of clinical experience who have completed standardized protocol training. Treatment will commence within 2–4 days of the onset of a spontaneous menstrual period following enrollment. The intervention lasts for 12 weeks, with sessions administered three times per week (every other day), totaling 36 sessions.

#### 2.7.1 Manual acupuncture group

The Manual Acupuncture (MA) group will receive treatment using sterile, single-use stainless steel needles (Hwato brand; 0.25 mm diameter needles in 25 mm or 40 mm lengths, and 0.30 mm diameter needles in 75 mm length). The details of acupoint locations and needling techniques are presented in **Table 2**. Treatment alternates between two fixed acupoint sets, commencing with Set A. Set A (supine position) comprises CV12 (Zhongwan), KI16 (Huangshu), CV4 (Guanyuan), KI12 (Dahe), EX-CA1 (Zigong), ST36 (Zusanli), and SP6 (Sanyinjiao). Set B (prone position) includes BL23 (Shenshu), BL33 (Zhongliao), and KI3 (Taixi). Based on the participant’s baseline TCM pattern diagnosis, which is categorized into four distinct patterns, two adjunct points will be added per session according to the following predefined protocol: (1) Kidney Deficiency Pattern: KI6 (Zhaohai) and LR3 (Taichong) (supine); GV4 (Mingmen) and BL18 (Ganshu) (prone); (2) Phlegm-Dampness Pattern: ST40 (Fenglong) and SP9 (Yinlingquan) (supine); BL20 (Pishu) and BL21 (Weishu) (prone); (3) Blood Stasis Pattern: LI4 (Hegu) and SP10 (Xuehai) (supine); BL15 (Xinshu) and BL17 (Geshu) (prone); (4) Liver Qi Stagnation Pattern: LR5 (Ligou) and LR3 (Taichong) (supine); BL18 (Ganshu) and BL19 (Danshu) (prone).

With the patient in an appropriate position, the local skin will be routinely disinfected. BL33 (Zhongliao) is inserted obliquely inferioromedially to a depth of 2.0-2.5 cun to target the third posterior sacral foramen. All other acupoints will be perpendicularly inserted to a standard depth of 1.0-1.2 cun. All needles will be manually manipulated to elicit the characteristic de qi sensation (a composite sensation of soreness, numbness, distension, or heaviness at the acupoint). All acupoint areas are disinfected with 75% alcohol swabs before needling. Needles will be retained for 20 minutes.

#### 2.7.2 Sham acupuncture group

The Sham Acupuncture (SA) group will receive treatment at predefined non-acupoint (NA) locations using sterile, single-use stainless steel needles (Hwato brand; 0.25 mm diameter, 25 mm length). The locations of these sham points, which are near but distinct from conventional acupoints, are specified in **Table 3** (e.g., 1 cun lateral to CV4).

**Table 3:** Intervention protocol for SA group.

Component	Acupoint / sham point name	Location	Needling details
Set A (Supine)	NA-1	1.0 cun lateral to the left of CV12.	Needle: Φ0.25×25 mm sterile needle. Depth: Superficial insertion of 2–3 mm.
	NA-2	1.0 cun lateral to KI16.	Same as above.
	NA-3	1.0 cun lateral to the right of CV4.	Same as above.
	NA-4	1.0 cun lateral to KI12.	Same as above.
	NA-5	10 mm lateral to ST36.	Same as above.
	NA-6	Midpoint between SP6 and the medial border of Achilles tendon.	Same as above.
Set B (Prone)	NA-7	Midpoint between BL23 and the inner line of Bladder Meridian (approx. 0.75 cun lateral).	Same as above.
	NA-8	1.0 cun lateral to BL33.	Same as above.
	NA-9	Anterior border of Achilles tendon, level with KI3.	Same as above.

Treatment will alternate between two standardized sets of sham points per session, starting with Set A (supine position), which comprises non-acupoints near CV12 (NA-1), KI16 (NA-2), CV4 (NA-3), KI12 and EX-CA1 (NA-4), ST36 (NA-5), and SP6 (NA-6). Set B (prone position) includes non-acupoints near BL23 (NA-7), BL33 (NA-8), and KI3 (NA-9). No pattern-based adjunct points will be applied.

With the patient in a suitable position, the skin at each sham point will be routinely disinfected. All sham points receive identical alcohol disinfection before needling. A needle will be inserted superficially to a depth of 2–3 mm. No needle manipulation will be performed, and no de qi sensation will be sought or elicited. The needle will be retained for 20 minutes.

Non-acupoint shallow needling was selected as the sham control to provide a credible blinding experience without specialized devices, while minimizing specific therapeutic effects by avoiding de qi and deep insertion. To minimize unblinding risk, the insertion depth (2–3 mm) and needle type were standardized across all sham sessions. All participants are informed that “acupuncture sensations may vary from person to person” to reduce expectation bias.

### 2.8 Concomitant medication

To ensure patient safety and standardize clinical management during the trial, the use of concomitant medication will be permitted and recorded under the following specific circumstances:

1. Rescue Medication for Prolonged Amenorrhea: If a participant experiences amenorrhea for more than 90 days during the trial period, oral dydrogesterone (10 mg, once daily for 10 days) may be administered to induce withdrawal bleeding. All rescue interventions will be thoroughly documented within the designated rescue medication section of the case report form (CRF).
2. Pre-existing chronic medications unrelated to gynecology or fertility management (e.g., agents for hypertension, hypothyroidism) may be continued throughout the trial, provided the site investigator confirms no interference with trial outcome evaluation. Full details of all concurrent medications—including drug name, dosage, administration frequency, and clinical indication—must be recorded comprehensively in the Concomitant Medication module of each participant’s case report form (CRF).

### 2.9 Outcome measurement

All outcome measurements will be scheduled according to the study timeline (**Table 1**). Baseline assessments will be performed before randomization and prior to the first treatment. Primary and secondary outcomes are assessed at the end of the 12-week treatment period (Week 12) and at the end of the 12-week follow-up period (Week 24).

#### 2.9.1 Primary outcome

The primary outcome is the cycle ovulation rate (COR). To account for the inherent irregularity of menstrual cycles in PCOS, the 12-week treatment period is divided into three standardized 28-day (4-week) time windows (i.e., Weeks 1-4, 5-8, and 9-12), each serving as an observation unit for detecting ovulation. COR is calculated using the following formula: COR (%) = (Number of 28-day time windows with ultrasound-confirmed ovulation/Number of completed full 28-day time windows) × 100% (19, 20). For participants who complete the full 12-week treatment period, the denominator is 3. For participants who withdraw early, the denominator is the number of full 28-day time windows completed before withdrawal. Participants who complete at least one full 28-day time window will be included in the primary analysis. Ovulation is confirmed by transvaginal ultrasound (TVUS), which serves as the gold standard. A window is considered ovulatory when TVUS documents the development of a dominant follicle (≥18 mm in diameter) followed by its subsequent collapse or disappearance, accompanied by the appearance of free fluid in the pouch of Douglas (29). Given that women with PCOS often have prolonged and irregular menstrual cycles, urinary LH test strips will be used daily to optimize the timing of ovulation monitoring. For each menstrual cycle, testing will begin on day 10–12 of that cycle and continue until ovulation is confirmed or the cycle ends. Once a positive LH test is observed, transvaginal ultrasound will be performed to confirm follicular rupture and ovulation. For participants who do not achieve a positive LH test, daily LH test strip use will continue until the end of that menstrual cycle. Daily basal body temperature (BBT) charting will be maintained as a supplementary record.

#### 2.9.2 Secondary outcomes

Menstrual cycle characteristics will be evaluated using two measures: 1) mean menstrual cycle length, calculated as the total number of days in the observation period (84 days for the 12-week treatment period, and 84 days for the 12-week follow-up period) divided by the number of menstrual cycles that occurred during that period. A menstrual cycle is defined as the interval from the first day of one menstrual bleeding to the day before the next menstrual bleeding. If no menstrual bleeding occurs during the entire observation period, the mean cycle length is considered missing, and the participant will be classified as having amenorrhea for that period. This value will be derived from participant-maintained calendars, and the average during treatment and follow-up will be compared to the self-reported baseline.; and 2) the menstrual cycle recovery rate, defined as the proportion of participants achieving a normalized cycle length of 21–35 days.

The sex hormone profile, measured in the early follicular phase (days 2–5 of a spontaneous cycle), includes the Free Androgen Index (FAI) (calculated as [total testosterone/sex hormone-binding globulin (SHBG)] × 100) (30) and the LH/FSH ratio.

The metabolic profile comprises three domains: 1) Anthropometric measures: body weight, height (for BMI), waist circumference (WC), and hip circumference (HC) to calculate the waist-to-hip ratio (WHR); 2) Glucose metabolism: fasting plasma glucose (FPG) and fasting insulin (FINS), with insulin resistance estimated by the Homeostatic Model Assessment index (HOMA-IR), a marker of insulin resistance, is calculated as (FINS × FPG)/22.5 (31); 3) Lipid profile: Total cholesterol (TC), triglycerides (TG), high-density lipoprotein cholesterol (HDL-C), and low-density lipoprotein cholesterol (LDL-C).

#### 2.9.3 Questionnaire measurement

Psychological well-being and quality of life are critical domains affected by PCOS and are important targets for holistic treatment evaluation. To comprehensively assess these patient-centered outcomes, the following validated instruments will be administered. The modified Polycystic Ovary Syndrome Questionnaire (MPCOSQ) (32): a 30-item scale evaluating health-related quality of life across seven domains: emotional disturbances, hirsutism, weight concerns, infertility distress, acne lesions, menstrual irregularity, and menstrual predictability; higher total scores indicate superior quality of life. Self-rating anxiety scale (SAS) (33) and self-rating depression scale (SDS) (34): a 30-item scale evaluating health-related quality of life across seven domains: emotional disturbances, hirsutism, weight concerns, infertility distress, acne lesions, menstrual irregularity, and menstrual predictability; higher total scores indicate superior quality of life. Patient global impression of change (PGIC): single-item 7-point Likert scale ranging from 1 (“very much improved”) to 7 (“very much worse”) to capture participants’ global subjective assessment of symptom change since trial initiation.

#### 2.9.4 Blinding assessment

Immediately after the final treatment session (within 5 minutes prior to post-treatment outcome assessments), all participants complete a masking evaluation. Participants are asked a single binary question: “Do you believe you received the authentic acupuncture intervention?” with three response options: “Yes”, “No”, or “Uncertain”.

#### 2.9.5 Safety outcomes

All adverse events (AEs) will be actively screened and documented at every visit through physician inquiry and patient self-report. This includes common acupuncture-specific AEs (e.g., local bleeding, hematoma, pain, bruising, fainting, or prolonged post-needling sensation) and general AEs (e.g., dizziness, nausea). The causality, severity, duration, management, and outcome of each AE will be documented in the Case Report Form. A Serious Adverse Event (SAE) is defined as any event that is fatal, life-threatening, requires hospitalization, or results in significant disability, and must be reported to the principal investigator and ethics committee within 24 hours of awareness. The frequency and severity of all AEs will be compared between MA and SA arms at trial completion.

### 2.10 Data management

All the trail data will be initially recorded on paper-based Case Report Forms (CRFs) at each site and subsequently entered into a secure, web-based Electronic Data Capture (EDC) system by trained coordinators following a specific data entry guide. The data quality is ensured through programmed range/logic checks within the EDC system, manual medical review by a clinical research associate (CRA) — including 100% source data verification for the primary outcomes — and regular audits by a dedicated data manager. All modifications to the database are logged with a timestamp, user identification, and a reason for change. Prior to the database lock, a comprehensive data management report will be prepared, reviewed, and approved by investigators and statisticians in a data review meeting to resolve queries and finalize the analysis dataset. The locked database, in SAS format along with its structure documentation and codebooks, will be delivered to the statistician for analysis. All study documents will be kept confidential by the lead research unit. Investigators and the ethics committee may access individual medical records as permitted by law, but any public reports will strictly protect participant anonymity, with every effort made to safeguard personal health information within legal bounds.

### 2.11 Quality control

To ensure protocol adherence and data integrity across all six centers, a multi-layered quality control system will be implemented. This system encompasses centralized, mandatory training and certification for all study personnel prior to the site initiation, pre-initiation site visits to confirm operational readiness, and routine, independent on-site monitoring to verify source data, informed consent, and protocol compliance. Furthermore, random fidelity checks of audio/video-recorded acupuncture sessions will be conducted by an independent expert to assess treatment adherence. The overall trial conduct, safety data, and protocol amendments will be overseen by an independent steering committee comprising clinical, methodological, and acupuncture experts.

### 2.12 Sample size

The sample size calculation was based on the primary outcome, the cycle ovulation rate (COR). Based on a systematic review of prior studies (15, 19, 20) and our pilot data[Trial Registration Number: ChiCTR2300073494], we anticipated a COR of 65% in the MA group and 40% in the SA group at the end of the treatment. Using a two-sided Chi-square test with a significance level (α) of 0.05 and a power (1-β) of 80%, the required sample size per group was calculated to be 62 participants. Accounting for an estimated 20% dropout rate over the 24-week study period, the final target sample size was 78 participants per group, for a total of 156 participants. This calculation was performed using PASS software (version 15.0).

### 2.13 Statistical analysis

All analyses will adhere to the Intention-to-Treat (ITT) principle. The baseline characteristics will be summarized descriptively by group using means (standard deviations) or medians (interquartile ranges) for continuous variables and frequencies (percentages) for categorical variables to assess between-group comparability.

For the primary outcome, the cycle ovulation rate (COR) at Week 12, any missing data will be handled using multiple imputation by chained equations (MICE) with 20 imputed datasets, and results combined using Rubin’s rules (35). The COR will be compared between groups using a linear mixed-effects model with study center as a random effect. The result will be presented as the mean difference between groups with its 95% confidence interval. As a sensitivity analysis, a per-protocol (PP) analysis will be performed, including only participants who completed the 12-week intervention with adequate adherence (defined as receiving ≥80% of the scheduled acupuncture sessions) and without major protocol violations.

To assess the sustainability of the treatment effect, the COR at Week 12 and Week 24 will be analyzed together using a linear mixed-effects model for repeated measures, including group, time point (Week 12, Week 24), and group-by-time interaction as fixed effects, with study center and participant as random effects (36).

The analyses for secondary outcomes will be conducted using available data. Continuous outcomes will be analyzed using Analysis of Covariance (ANCOVA), adjusting for corresponding baseline values and study center (37). Binary outcomes will be analyzed using generalized linear mixed models, incorporating study center as a random effect (38). All statistical tests will be two-sided, and a p-value < 0.05 will be considered statistically significant. All analyses will be performed using SAS software (version 9.4, SAS Institute Inc., Cary, NC, USA).

## 3 Discussion

This protocol details a prospective, multicenter, randomized, sham-controlled trial designed to evaluate the efficacy of manual acupuncture for improving ovulation in a precisely defined subgroup of women with polycystic ovary syndrome (PCOS). Adhering to international guidelines (CONSORT, STRICTA, SPIRIT), the trial addresses key methodological limitations of previous studies through a credible sham control, central randomization, stringent blinding, and a pre-registered, objectively defined primary endpoint (ultrasound-confirmed ovulation). The sample size (156 participants) is adequately powered to detect a clinically meaningful difference in cycle ovulation rate between groups. A central and innovative design feature is the strategic refinement of the study population via stringent criteria. This approach aims to reduce confounding from clinical heterogeneity, thereby providing a clearer test of the intervention’s specific efficacy and enhancing the interpretability of the treatment effect.

Central to the intervention is a standardized yet flexible acupuncture protocol. It is built upon the TCM principles of tonifying the Kidney and regulating the Chong and Ren meridians, implemented through a fixed core set of acupoints (e.g., CV4, BL23, SP6, KI3) for consistent treatment. This core is then supplemented with adjunct points to accommodate individual TCM pattern presentations in PCOS, striking a balance between methodological rigor and personalized clinical application. This active intervention is compared against a sham acupuncture procedure employing non-acupoint, shallow needling, which was designed to provide a credible control for non-specific effects while maintaining participant blinding. This sham procedure was selected to balance methodological rigor with practical feasibility across the six participating centers.

Central to the intervention is a standardized yet flexible acupuncture protocol, built upon the TCM principles of tonifying the Kidney and regulating the Chong and Ren meridians. It is implemented through a fixed core set of acupoints (e.g., CV4, BL23, SP6, KI3) for consistent treatment, supplemented with adjunct points to accommodate individual TCM pattern presentations in PCOS. This design strikes a balance between methodological rigor and personalized clinical application. The active intervention is compared against a sham acupuncture procedure using non-acupoint, shallow needling, designed to provide a credible control for non-specific effects while maintaining participant blinding. This sham procedure was selected to balance methodological rigor with practical feasibility across the six participating centers.

Beyond these general quality measures, the primary methodological innovation is the proactive management of clinical heterogeneity. Recognizing that PCOS encompasses distinct phenotypes with potentially divergent treatment responses, this trial applies stringent inclusion criteria. By enrolling only women with spontaneous menstrual cycles of 35–60 days and excluding those with a BMI ≥28 kg/m², we aim to create a more homogeneous cohort characterized by mild-to-moderate ovulatory dysfunction. This deliberate population refinement seeks to minimize phenotypic confounding and provide a clearer signal of the intervention’s specific efficacy.

Our population selection was guided by methodological considerations to enhance internal validity. Women with BMI ≥28 kg/m² were excluded according to the Chinese obesity criteria (37), and those with spontaneous menstrual cycles of 35–60 days were enrolled based on the definition of irregular menstrual cycles in the 2023 International PCOS Guideline (4), defining a subgroup with mild-to-moderate ovulatory dysfunction.

We acknowledge several limitations. The sham acupuncture procedure may not be completely inert and that sensory differences between the two groups could theoretically lead to unblinding. However, we have implemented standardized procedures (uniform insertion depth, no manipulation, and standardized patient communication) to minimize this risk. However, this conservative design strengthens the validity of any positive finding. The stringent inclusion criteria, while methodologically justified for this proof-of-concept study, will limit the generalizability of the findings to the broader PCOS population, particularly women with the obesity subtype or more severe anovulation. Furthermore, the inability to blind acupuncturists is an inherent constraint in acupuncture trials; we have sought to mitigate this performance bias by standardizing all other aspects of patient interaction and maintaining strict blinding of participants and outcome assessors. Additionally, this trial does not include long-term reproductive outcomes such as clinical pregnancy rate or live birth rate, which will require future investigation.
