# Supplementary material for: Acupuncture for ovulation induction in polycystic ovary syndrome: study protocol for a multicenter, randomized, sham-controlled trial
Source: Front Endocrinol (Lausanne). 2026 Aug 5;17:1893429. doi: 10.3389/fendo.2026.1893429 (PMC13485508; doi:10.3389/fendo.2026.1893429)
Supplement: Supplementary file 1 [file Table1.docx]

Supplementary Material

# 1 Supplementary Table

| **Supplementary Table 1. TCM Pattern Differentiation Criteria for PCOS** | | |
| --- | --- | --- |
| **Pattern** | **Main Symptoms**  **Secondary Symptoms** | **Tongue and Pulse** |
| Kidney Deficiency | Oligomenorrhea, dizziness and tinnitus, lumbosacral soreness and weak knees, decreased libido, scanty or absent vaginal discharge, vaginal dryness and pain | Pale or dark tongue, white coating; deep, thready, or weak pulse |
| Phlegm-Dampness | Oligomenorrhea, obesity, profuse white and sticky vaginal discharge without odor, dizziness and palpitations, chest tightness and nausea, facial puffiness, fatigue and heaviness | Pale or enlarged tongue with thick, greasy white coating; slippery or soft pulse |
| Blood Stasis | Oligomenorrhea, rough, dry, and scaly skin (resembling fish or turtle shell), dysmenorrhea with fixed stabbing pain relieved after clot passage | Dark red or purplish tongue with petechiae or ecchymoses; choppy or wiry pulse |
| Liver Qi Stagnation | Oligomenorrhea, depression or mood swings, frequent sighing, chest and hypochondriac fullness or distending pain, lower abdominal distending pain, premenstrual breast distension, premenstrual mood changes | Pinkish or purple tongue with thin white coating; wiry pulse |
